# A Common Cold

**Published:** 1909-12-18

**Authors:** Seymour Taylor

**Affiliations:** Senior Physician to the West London Hospital. (A Lecture of the West London Hospital Post Graduate Course)


### Hospital Clinics.

### A COMMON COLD.

By SEYMOUR TAYLOR, M.D., F.R.C.P.; Senior Physician to the West London Hospital.

(A Lecture of the West London Hospital Post Graduate Course.)

Probably the most frequent ailment in this country of ours is the one known as " a cold "; and although in the majority of instances the ailment is of a mild character, which may be, and usually is, left to run its course, in other instances the disease is severe in its symptoms, and may be far-reaching in its effects. Further, the amount of misery, though often small in the individual, is so extensive when the multitude are considered, and the waste of time and money is so enormous, that I have thought this subject is not one beneath our consideration.

If I am asked to define an ordinary " cold," I should say that it is an acute, febrile, specific infectious and contagious disease probably produced by many micro-organisms, characterised by catarrh of the respiratory tract and its off-shoots, accompanied by sneezing, with discharge from the nose and bronchial mucous membrane. A definition of the disease is important, so that we can accurately know what malady we are discussing. It is a specific fever, a " heat " rather than a cold, which is eminently " catching "; and the sooner the public know, and the sooner our authorities act on this fact, the better it will be for the benefit of the community. When a lady says to her friend, " Do not come to our house at present, the children have mumps," she recognises the infectious character of the disease, and she speaks sound prophylaxis. And where another says, " Do not come near me, for I have a bad cold," she is equally wise in her precaution, though the advice is not always followed. Yet I have no hesitation in saying that according to my experience no one of the infectious diseases is more likely to spread in a house or in a community than is a " cold." At certain seasons, when colds are rife, it is not so much that a large community is at once stricken with the disease, but it is rather that the disorder, having attacked some individuals, they, either by their own carelessness, or the want of precaution on the part of others, become active foci of infection, from which the disease extends to, or is taken by, others, until after a few days a large epidemic exists.

Let me relate a typical example. A little boy (M.) returned to school in perfect health after the summer holidays. On the fourth day of the new term two brothers came to school with typical symptoms of " cold," with the accompanying sneezes, sniffings, lachrymation, and cough. They occupied desks, one on each side of M. In twenty-four hours M. is down with a severe form of naso-respiratory catarrh, and has to be away from school six weeks. During the first two days of his illness two cousins, N. and N., are inadvertently allowed to visit him and stay for tea. These two are taken, in about thirty hours, with precisely the same symptoms as ushered in M.'s attack. Thereupon the disease spreads to every member of the house in which N. and N. live, attacking mother, father, brothers, and sisters and the domestic servants. One of these latter visits her friends at Croydon, some twelve miles away, and it is subsequently ascertained that the whole household is sick in turn with " severe colds," in from two to six days. And so, from this first infectious centre, the disease was no doubt spread and propagated.

### Ætiology.

Some families are especially prone to acquire the disease. Amongst the juveniles it will be more rife, epidemics will be more frequent, and individual attacks more severe, with those members of the family who have naso-pharyngeal adenoids, or whose naso-respiratory mucosa is in an unhealthy state. This is what one would expect when we bear in mind that the specific micro-organism or organisms are most probably received by, and inoculates the system at, the lower nasal passages. But in adults, even, with healthy mucous membranes there are some individuals, and even families, who are especially prone to acquire the disease, just as there are others who invariably have attacks of hay-fever in the summer months, even though they be town-dwellers.

The disease confers no immunity from subsequent attacks. On the contrary, it rather predisposes the individual sufferer to subsequent invasions. This is important as bearing on the risk of exposure of delicate children and adults. Some people are always " catching colds." I know families no member of which can come in contact with anyone who has a cold without his or her contracting the disease, and thus spreading it to the other members. Further, it often is found in such susceptible families that first recrudescences or relapses occur during convalescence, as in typhoid fever. Such victims are said to have " renewed their colds," or to have " caught a fresh cold." The probability is that they have received a fresh invasion of the poison.

The disease is most frequent in young adults, though no age is exempt. I have seen it occur in the babe and in the octogenarian. The duration of the fever varies; there is no fixed time for it to run its course, as in (say) scarlet fever. But a marked difference is observed in the power of recovery. In youth a cold rarely lasts longer than five or six days, whilst in elderly people it may run a course of ten days or more, and one often hears the expression, " I cannot throw off my colds as I formerly did."

The intensity of an individual attack, or the severity of an epidemic, varies. In other words, the virulence of the poison varies in different epidemics and in various individuals. Two factors are here probably at work, namely, the varying potency of the poison and the prepared receptivity of the sufferers. Speaking on this last point I have observed that those people who, being prone to catch a cold, are overworked, depressed, or generally run down, are the earliest and most severely affected of the victims. But it is well known that this lowered vitality is a strong predisposing factor. The man or woman who carelessly leaves off a top coat or outer wrap and comes in contact with the poison, will run a risk of taking the fever; but it is chiefly owing to his or her vitality or resisting power being diminished by the bodily temperature being lowered. A "thorough draught," that frequent bug-bear of the public, probably means an imperfectly ventilated room or hall. The colds which people catch as a consequence of exposure to this draught are not due to the blast of fresh air of a lower temperature than that which they have been inhaling, but are probably consequent on the lowered vitality caused by discarding an outer garment on entering the hall or church. The victims are probably inhaling the poison all the time, and many will acquire the disease, wraps or no wraps, draught or no draught; but the suddenly lowered temperature of the body is the culminating causal factor.

Let the bodily temperature be maintained by suitable garments and no draughts will of themselves cause the disease. The engine driver, the bargeman, the cab driver are all exposed to the gales of rude Boreas, but one seldom hears of them catching colds. On the other hand, how often is the disease traced to exposure at the graveside, with its attendant lowered nervous vitality. Again, let anyone enter a public hall or a church or chapel immediately after the congregation has left, and he will at once be struck by the unhealthy character and the considerably higher temperature of the atmosphere inside the building as compared with that outside. The poison has been acquired within the walls; it is brought to fruition by bodily exposure during the journey home.

Season of the year has an important bearing on the outbreak of an epidemic. In my experience the most frequent epidemics occur in early autumn, about the end of September, when the heat of the sun's rays is diminishing, and dew begins to condense on the surface of the ground. One notices about this time of the year that the ventilating shafts which open into the roadways are frequently emitting an obvious steam-like vapour. That is the period when I find, from my records, that colds are most rife. People have returned to town from their holidays, the children go to school, and the parents resume their usual avocations. Yet how frequently is it our experience to find that whole families or even schools are afflicted with colds within a week after their return from the seacoast or the moor? I have a firm opinion, based on my own personal experience, as well as that of others, that the emanations from the urban drains contain the poison or poisons which cause these autumnal epidemics. I have not the time, even had I the ability, to investigate these sewer gases; but I have records of numerous colds, of a very virulent type, which have suddenly supervened within forty-eight hours after passing over one of these ventilating shafts and inhaling the escaping vapours. I know of two or three families who invariably "catch colds" towards the end of September, and my own personal experience is the same. There are one or two ventilating apertures which in this season of the year are veritable traps, and I am a victim of a severe cold within twenty-four hours of my exposure if I inadvertently pass them.

The exciting cause of "cold" is a specific micro-organism. It is quite possible, nay probable, that, as in pneumonia, more than one particular bacterium may cause the disease; and it is equally probable that in widespread epidemics some of the various bacteria which give rise to cold are spread abroad, and are at work on the community. Here is a list of micro-organisms which may be, and often are, found in the nasal cavities and the discharges therefrom, when a patient is suffering from severe cold: Micrococcus catarrhalis, streptococci, staphylococci, micrococcus tetragenus, diplococci, and a special coccus occurring in tetrads and large masses (Benham).

Possibly, from what I have read, this last may be the most potent producer of "colds." The evidence which I have been able to sift convinces me that the specific organism obtains its entry to the system through the mucous membrane of the nose, and occasionally that of the mouth. That is to say, the disease is air-borne, and should it enter by the mouth it is through improper inhalation with open mouth, and not by food. Now, if this be granted, it is obvious that the sooner the public recognises that a cold is a catching, and also a preventable disease, the better for the comfort, the health, and happiness of the community.

### Symptoms.

The onset of the disease may be quite sudden, and usually is. The victim, if an adult, may return home from work and remark that he feels that he has caught cold. In my own case, when I have been exposed, I may be able to perform my work for the next twenty-four hours in comfort; but suddenly, at about the end of that period, a sense of general discomfort supervenes. There are head aches more or less severe, shiverings, thirst, a dry skin, and, in short, the general feeling of malaise which is common to many other specific fevers. But there is one usual and almost constant symptom. The patient complains of dryness and discomfort at the back of the nose; or there is a sense of burning which appears to occupy a small area of the upper and posterior surface of the soft palate. This spot would appear to be the starting point of the inflammatory invasion; and from this centre the dryness or heat appears to extend forwards to the anterior nares, or backwards over the soft palate, or upwards to the higher nasal cavities and its offshoots, to the frontal sinuses and to the lachrymal ducts. And in bronchitis is usually spreads over the epiglottis (both sides) and down to the larynx, trachea, and the upper divisions of the bronchial tree. Within a few hours the respiratory tract and its offshoots are then in the earlier stages of a specific inflammation, and the victim's distress is acute, the distress being chiefly aggravated by a sense of extreme dryness and heat referred to these parts. The temperature is increased—often going up to 100° F., or higher—and the pulse-rate approximates 90. This stage of invasion is the most distressing. Many nostrums are advertised with a promise for its alleviation. Soon, however, in a few hours at the longest, the mucous membranes of the invaded tracts and passages pour out a secretion, abundant and clear at first, and occasionally acrid.

There is sneezing, lachrymation, and a frequent demand for the pocket handkerchief. Then, after another twenty-four hours, the nasal discharge becomes muco-purulent, and later on purulent, with lessened discomfort. If the tracheal or bronchial mucosa is involved, the patient has cough with expectoration, at first glairy and tenacious, and subsequently, during convalescence, purulent. With this the fever as a rule gradually subsides, and convalescence sets in. Complete cure will, however, be speedily or slowly attained according to the varying circumstances of environment, constitution, and treatment.

Occasionally, these symptoms are complicated with intense headache, and pains in the limbs, loins, and back, as though a double infection, influenza and catarrh, had invaded the system. But we have no proof of this. The accounts of severe colds written thirty years ago, when there was no influenza epidemic, describe cases with similar general pains. If these pains accompany a severe cold, we must in default of unmistakable evidence of influenza regard them as manifestations of the severity of the catarrhal invasion. It is necessary also to remind you that many cases which the sufferers themselves diagnose as "colds with influenza" are either cases of alcoholism or are ordinary colds which they make an excuse for alcoholic indulgence.

### Complications.

The immediate complications of the disease are for the most part due to extensions into the various cavities and tubes opening into the nasal fossæ. Thus we may meet with pains in the superior maxillæ, old carious teeth are found out, and dormant toothache aroused, or even abscess of the antrum may supervene. The lachrymal ducts may be obstructed or inflamed, or they may suppurate. Middle-ear deafness is not uncommon. Adenoid vegetations become more swollen, and laryngitis or tracheitis may give rise to alarming symptoms. Then, as more remote effects, one may trace many cases of permanent deafness, or stenosis of the lachrymal ducts, and even phthisis, to the effects of previous severe or continued colds.

### Treatment.

In a large number of cases the public treat themselves. They either neglect the cold, and let it run its course, or they prescribe, on their own authority, ammoniated tincture of quinine, or eucalyptol, or some simple household remedy. Others buy lung tonics, balsams, syrups, and such like useless, but possibly harmless, quack remedies. If the sufferer from a severe cold will only remain in bed, or keep in a warm, well-ventilated room, and take liquid nourishment, with a plentiful allowance of oranges, and other juicy fruits, with frequent libations of barley water, linseed tea, toast water, and the like, he will be agreeably surprised at the speedy relief which he obtains, and the short course of his attack. But if he will neglect these precautions, or if the necessity for obtaining a livelihood compel him to pursue his avocation, then he must expect a longer duration of his illness, and the probable risk of renewed or fresh invasion of the fever.

I know of no remedy with which it is possible to cut short, or abort a cold, unless it be formalin. The disease will run its course; but in some the duration is shorter than in others. Besides environment, and palliative treatment, we must recognise that some individuals and some families are specially prone to acquire colds of a severe or virulent type, and in these instances palliative treatment has very little effect, and no medicinal remedies, so far as I know, have any appreciable effect in cutting short the fever. But, on the other hand, no matter however severe be the type of the fever, we find remedies which are of immense service in preventing the spread of the inflammatory process to other parts of the respiratory track, and so lessening the chances of grave complications and sequelæ. Suppose then that we have a free hand in the treatment of a patient who has a severe cold, what line of treatment is best to adopt?

The following general rules should be observed. Keep the patient in bed in a well-ventilated room, with windows open top and bottom, and, if necessary, a brisk fire burning. Extra clothes can be put on the bed if the patient complains of the draught. All forks, spoons, and feeding utensils should be kept apart from those of the household; and they should be washed in some antiseptic fluid. In short, you should adopt those methods in the general management of the bedroom which you would carry out in any other form of infective fever, and these measures may be included in the words "medical Listerism."

The comfort of the patient will be considerably enhanced by anointing the nose and lips with some simple unguent in which bi-carbonate of soda is the principal ingredient. This relieves the discomfort during the first, or dry, stage of the disease, and also soothes the nostrils and lips when they would otherwise become excoriated by the acrid nasal discharge. The ointment should be applied freely, and even slightly rubbed into the skin; and it may with great advantage be smeared or painted with a camel hair brush inside the nostrils. The greased rag of our grandparents was not an approved remedy unless its reputation had been founded on years of tradition with beneficial experience.

I find that irrigation of the nasal passages—best by a simple rubber tube used as a syphon—is of immense relief. A ball syringe can be used if preferred. Children soon acquire the knack of using a nasal douche, and when they have once experienced the relief which it affords they gladly employ the remedy. The irrigating fluid may be simple warm water, or, better still, an alkaline solution (bicarbonate of soda gr. x. to the ounce), by which the acridity and the temporary acidity of the nasal discharges are diminished and neutralised. I am not in favour of snuffs. Though the active ingredient of these local medicating powders may be cocaine, or morphine, or menthol, the admixture of powdered gums as vehicles causes discomfort. True these snuffs dry up the secretions, but in so doing they leave hard and irritating crusts, which are a source of some discomfort and annoyance. If it be considered advisable to apply any one of these remedies to the nasal cavities they had better be in the form of an ointment, or of a douche.

As regards internal remedies, we must remember that there are two sets of symptoms calling for relief—namely, those due to nasal irritation and those due to the constitutional disturbance. In my experience salicin and the salicylates, especially if freely administered, will quickly relieve the headaches and the pains, which are often distressing, and will also bring down the fever. But I have no recollection of a single case in which this drug or its compounds exerted the slightest influence on the nasal catarrh as regards its abbreviation.

A far more efficacious remedy is morphine, and you may, notwithstanding the textbooks, give it to children and to adults. It is only a question of dosage. Children can take $\frac{1}{25}$ gr. three or four times a day without inconvenience. Adults may take $\frac{1}{12}$ gr. every four hours. The remedy may be given with liq. ammoniæ acetatis and oxymel scillæ, and by this combination we obtain not only free and comforting diaphoresis, but an easier expectoration.

Salol as a local disinfectant is of some value. It relieves the faucial congestion, and, if given at all, it should be prescribed in the earliest stage of the cold. Otherwise, the fever having obtained a good start, it is not, in my experience, of any avail in the direction of cutting short the disease. It is best given in the form of spray to the mouth, pharynx, and the nasal cavities.

Formalin again is a most valuable local remedy; it arrests the disease, or, at least, shortens its duration. In my experience it is best applied in the form of a spray (1 per cent.), combined with some oily substance, such as purified paraffin. As the two substances mingle with difficulty, it is necessary that the mixture should be well shaken up before spraying the nose. Or it may be incorporated with a purified paraffin (ateolin), supplied by Messrs. Bell and Croyden. This elegant preparation can be applied to the nostrils, within and without, and to the lips, by means of a camel hair brush. I have recently seen excellent results. But I would again impress on you the advantage of some form of oil or grease as a vehicle for all local applications. Peroxide of hydrogen is also of some benefit so far as my limited experience of this remedy goes; but as it can only be applied as a douche it has its disadvantages. Quinine, unless in large, and therefore distressing, doses, has very little good effect.

